# Clinical presentation, outcome and prognostic factors in dogs with immune-mediated haemolytic anaemia: a retrospective single-centre study of 104 cases in Ireland (2002–2020)

**DOI:** 10.1186/s13620-024-00277-w

**Published:** 2024-07-25

**Authors:** Antoine A. Duclos, Esther López Bailén, Kathryn Barr, Kevin Le Boedec, Benoît Cuq

**Affiliations:** 1 Small Animal Clinical Studies, School of Veterinary Medicine, University College Dublin, Dublin 4, Ireland; 2 Langford Vet, University of Bristol, Langford, UK; 3 Internal Medicine Department, Centre Hospitalier Vétérinaire Frégis, Gentilly, France

**Keywords:** Haematology, Immunology, Anaemia, Hyperbilirubinaemia, Thrombocytopenia, Prognostic factors, Long-term outcome

## Abstract

**Background:**

Immune-mediated haemolytic anaemia (IMHA) has a high mortality rate within the first weeks to months of diagnosis. Identifying dogs at increased risk of death may help guide decision-making for owners and veterinarians. Prior studies have identified several but inconsistent prognostic factors. The objectives of the study were to describe the clinical presentation and outcome of canine immune-mediated haemolytic anaemia in Ireland and to assess for independent factors associated with survival including long-term survival. Medical records from a single centre were reviewed between 2002 and 2020 to identify dogs with immune-mediated haemolytic anaemia using the American College of Veterinary Internal Medicine (ACVIM) consensus statement algorithm. Survival analysis was performed using univariable Cox proportional hazards regression models with Breslow method for ties to identify prognostic factors.

**Results:**

One hundred and four cases were included. The diagnosis of immune-mediated haemolytic anaemia was classified as definitive, supportive and suspicious in 42 (40%), 50 (48%), and 12 dogs (12%) respectively. Twenty-two dogs (21%) were diagnosed with associative IMHA and 82 dogs were diagnosed with non-associative IMHA (79%). 65% of the cases received more than one immunosuppressive medication during the course of treatment. The mortality rate at one and three months was 16% (95% confidence interval [CI] 9–26) and 31% (95% CI 21–43) respectively. Excluding dogs that died within three months, the median survival time was 2664 days. The relapse rate during the follow-up period was 7%. Survival did not improve over the course of the study period. Thrombocytopenia and hyperbilirubinaemia were identified as negative prognostic indicators (Hazard ratio 2.2 and 2.5, 95% CI 1.1–4.1 and 1.1–5.6, respectively).

**Conclusions:**

Excluding dogs that died within three months, the outcome was good in dogs with non-associative immune-mediated haemolytic anaemia in Ireland. The relapse rate was low regardless of the presence of associative causes. Thrombocytopenia and hyperbilirubinaemia were the only independent negative prognostic factors. The one-month and three-month mortality rates were similar compared to prior studies and survival did not improve over time during the study period: the mortality rate of canine immune-mediated haemolytic anaemia remains high in the acute phase.

## Background

Immune-mediated haemolytic anaemia (IMHA) is a common haematological disorder in dogs [1]. The pathogenesis involves an immune response directed against self-antigens expressed on the surface of erythrocytes, leading to their destruction [2]. IMHA is associated with a high morbidity and mortality due to the often-profound anaemia, cost of management, potential requirement for multiple blood transfusions, potential for side effects or refractoriness to immunosuppressive therapy and predisposition to devastating thromboembolic events [3, 4].

Despite steady progress in understanding, diagnosing and managing canine IMHA, the short-term mortality remains high: 18–44% within the first two months in the largest and most recent studies; although historical studies report a higher mortality rate up to 70% [5–10]. In previous studies, among many other factors, thrombocytopenia, hyperbilirubinaemia and increased urea concentration were most consistently associated with a poor outcome [7, 9, 11–18]. IMHA is a complex disease and integrative illness severity scores such as the *Tokyo* and the Canine Haemolytic Anaemic Objective Scores (CHAOS) were developed, but have been so far inconsistently useful in predicting outcome [7, 11, 15, 19].

Survival analyses are rare in the veterinary literature and precede the ACVIM Consensus Statement for strict criteria of inclusion and classification of canine IMHA [6, 15]. Additionally, studies assessing specifically the long-term survival (defined as survival for more than three months) are scarce [10]. Finally, there are no data regarding the clinical presentation, long-term outcome and prognostic factors of immune-mediated haemolytic anaemia in a population of dogs from Ireland specifically.

The objectives of this study were to evaluate clinical presentation, clinicopathological findings, illness severity scoring system (CHAOS) and medical interventions as potential prognostic indicators for short- and long-term outcome of canine IMHA.

We hypothesized that the clinical presentation would be similar compared to prior studies, that the survival would have improved over the study period, and that the type of immunosuppressant used would be associated with increased survival. We also hypothesized that previously-identified prognostic factors such as increased urea concentration, thrombocytopenia and hyperbilirubinaemia would be independently associated with survival in this population.

## Methods

### Study design

The electronic medical records (VetSCOPE^®^; Lawler Developments Limited) from a single institution were searched for the words “immune-mediated haemolytic anaemia”, “immune mediated haemolytic anaemia”, “autoimmune haemolytic anaemia”, “autoimmune anaemia”, “immune-mediated anaemia”, “haemolytic anaemia”, “AIHA” or “IMHA” in the “diagnosis” field. Individual medical records were reviewed to identify all client-owned dogs diagnosed with IMHA between January 2002 and December 2020.

Using the ACVIM consensus statement [20], inclusion criteria at the time of presentation included the presence of anaemia (haematocrit < 0.37 L/L), evidence of haemolysis: hyperbilirubinaemia, haemoglobinaemia, haemoglobinuria, or presence of ghost cells on blood smear examination; and evidence of immune-mediated erythrocyte destruction: spherocytosis, positive Coombs’ test [1:16 and above] and/or positive slide agglutination test (SAT) with 1:4 dilution.

The likelihood of IMHA diagnosis (diagnostic, supportive, or suspicious) was retrospectively assessed from the records using the ACVIM consensus statement diagnostic algorithm [20]. When diagnosis was suspicious, several additional parameters were taken into account to determine inclusion. These parameters were the following: signalment, available investigations performed prior to referral, prior immunosuppressive therapy that may have affected results upon referral, complete work-up for exclusion of other causes of anaemia and follow-up documenting response to immunosuppressive treatment.

For all included dogs, the following information was collected: age, sex, neuter status, breed, body weight, medication prior to presentation (including immunosuppressants), and clinical signs with their duration. Haematology, blood smear review (performed either by a technician or by a clinical pathologist), SAT, Coombs’ test and biochemistry at the time of presentation had to be available for review. From the biochemistry, only urea, creatinine, bilirubin and albumin concentrations were recorded as these parameters were required to calculate the CHAOS score and were previously associated with survival in IMHA or non-regenerative immune-mediated anaemia [7, 15, 21].

The presence of associative conditions was assessed by review of the available body cavity imaging reports (thoracic radiographs and abdominal ultrasound or thoraco-abdominal computed tomography). Additionally, testing for vector-borne diseases (polymerase chain reaction PCR or serological testing for *Babesia spp*,* Ehrlichia canis*, *Anaplasma phagocytophilum*, and *Borrelia burgdoferi)* was performed depending on the travel history. *Angiostrongylus vasorum* blood rapid immunoassay was performed if the clinical presentation was deemed evocative and depending on the parasiticide treatment history. Urine culture was performed depending on the urinalysis results. For specific cases, additional investigation was performed at the discretion of the attending clinician and recorded when relevant (e.g., cytological examination of various organs including peripheral lymph nodes, liver, spleen and pulmonary nodules, echocardiography, post-mortem examination). Recorded treatment during hospitalisation included type and number of blood transfusions, immunosuppressants and anti-thrombotic drugs.

The CHAOS score was retrospectively calculated, using the previously published model [7]. The included cases were then categorized as associative or non-associative using the recent ACVIM consensus statement [20].

Thrombocytopenia was defined as having a platelet count below 150* × *10^9^/L with confirmation on blood smear examination. Severe thrombocytopenia was defined as having a platelet count below 50* × *10^9^/L. Non-regenerative immune-mediated anaemia (nrIMA) was defined as non-regenerative anaemia persisting for more than five days after presentation with an absolute reticulocyte count below 60 × 10^9^/L, signs reported to have lasted for three weeks of more prior to presentation, satisfaction of ACVIM criteria and response to immunosuppressant therapy. Performance of bone marrow cytology and histopathology was not mandatory for the cases to be included in this category. When performed, bone marrow examination had to document erythroid hyperplasia.

Regarding the outcome, time to death, cause of death (euthanasia or spontaneous death, IMHA-related death or non IMHA-related death), or time to last the follow-up were assessed. Deaths were attributed to IMHA when anaemia was not controlled despite immunosuppressive therapy, when marked clinical deterioration was identified and attributed to IMHA when thromboembolic events were documented. For the latter, the diagnosis was either suspected based on clinical presentation (e.g., acute respiratory distress for pulmonary thromboembolism and no other causes identified) or confirmed based on imaging or post-mortem findings. Relapses were defined by documentation of anaemia while investigating a new onset of clinical deterioration following documentation of control of clinical signs and anaemia on the previous assessments. Thorough investigations of the anaemia did not have to be performed for the cases to be considered as relapse of IMHA if the clinical condition was deemed evocative and if the dogs were documented to respond to recommencement/intensification of immunosuppressive therapy.

Cases with incomplete medical record or for which other causes of anaemia could not be ruled out were excluded. For the purpose of the survival analysis, the date and cause of death or the date of the patients’ last visit were obtained by contacting the referring veterinarian listed in the file when not available in the medical records.

The study design was approved by our institution Research Ethic Committee (AREC-E-21-02).

### Statistical analysis

The dogs were divided in three categories depending on their body weight (< 10 kg, 10–25 kg, > 25 kg). Given the extended study period, and to allow for investigation of possible variation of treatment and survival over time, the 1/3rd and 2/3rd percentiles of the variables year of diagnosis were used to divide the study period into 3 balanced categories (2002–2013, 2014–2018 and 2019–2020).

Data analysis was performed using a commercially-available statistical software (Stata^®^ StataCorp LLC version 17.0). Continuous data were presented as median and minimum-maximum. Categorical data were expressed as frequencies. Cox proportional hazards regression model was used to assess for independent factors for survival. Kaplan-Meier curves were obtained by assessing all-cause mortality. Dogs lost to follow up or alive at the end of the study were censored. Dogs with associative conditions were excluded from the survival analysis. Univariable Cox proportional hazards regression models with Breslow method for ties were used to screen for prognostic factors. All the variables with *P* < 0.2 on univariable analysis and all potential confounding variables were entered into the initial multivariable model. Then, the variable with the highest *P*-value was removed from the model and the performance of the new model was compared with the one of the previous models by likelihood ratio test. Backward elimination was used for model selection; variables were removed by ranking the clinical relevance, with the least relevant variable being removed first. Likelihood ratio tests were performed following removal of each variable to test variable significance to the model. The process was repeated until all the variables retained in the model were significant or the *P*-value of the likelihood ratio test became < 0.05. Hazard ratios (HR) were calculated and presented with 95% confidence interval (CI). For all Cox regression models, proportionality assumption was confirmed by the test of proportional-hazards assumption.

## Results

### Population

The records of 203 dogs were reviewed. Ninety-nine cases were excluded due to unavailable or incomplete medical records which did not allow to confidently rule out other causes of anaemia or determine associative conditions. The remaining 104 dogs met the inclusion criteria. A two-year-old dog that presented after receiving a blood transfusion just prior to referral was no longer anaemic on presentation. This dog was included based on available blood work prior to referral, supportive evidence for IMHA, lack of other causes of anaemia identified and satisfactory progression with immunosuppressive therapy upon follow-up.

Thirty-eight dogs were diagnosed between 2002 and 2013, 38 dogs were diagnosed between 2014 and 2018 and the remaining 28 dogs were diagnosed between 2019 and 2020. Sixty-eight of the ninety-nine excluded cases were diagnosed between 2002 and 2013.

The median age was 6 years (0.7–14). Out of the 104 dogs, there were 74 females (71%) and 30 males (29%). The neuter status was unknown in two dogs. Out of the 102 remaining dogs, 48 were female neutered (47%), 23 were female entire (22%), 10 were male neutered (10%) and the remaining 21 dogs were male entire (21%). The median weight was 16.2 kg (3.9–87.6). Thirty-one dogs weighed less than 10 kg (30%), 50 dogs weighed between 10 and 25 kg (48%) and the remaining 23 dogs weighed more than 25 kg (22%).

There were 38 breeds represented with crossbreed dogs being the most common (21 dogs). The most represented pure breed was the Jack Russell Terrier (10 dogs), followed by Shih Tzu [8], Springer Spaniel [8], Cocker Spaniel [7], Labrador Retriever (5 dogs), German Shepherd [4], Miniature Schnauzer [4], Border Collie [3], Newfoundland [3], Dachshund [2], Standard Poodle [2], Pomeranian [2] and Japanese Spitz [2] and one dog of each following breeds: Beagle, Bernese Mountain Dog, Bolognese, Bichon Frisé, Maltese, Boxer, Cairn Terrier, Cavalier King Charles Spaniel, Golden Retriever, Greyhound, Mastiff, Miniature Pinscher, Pug, Samoyed, Soft-Coated Wheaten Terrier, Staffordshire Bull Terrier, Vizsla and West Highland White Terrier.

### History and clinical signs

The median duration of the clinical signs was 4 days (1–90). One hundred and three out of 104 dogs presented with pallor (99%). The other common clinical signs were the following: 75 out of 104 dogs presented with lethargy (72%), 57 had inappetence (53%), 62 dogs were icteric (60%), 24 had pyrexia (23%), and gastrointestinal signs were relatively common with diarrhoea documented in 22 dogs (21%) and vomiting in 8 dogs (7%).

Treatments administered prior to referral were available for 91 dogs. Steroids were administered in 61 dogs prior to presentation (67%). Antimicrobials were administered in 51 dogs prior to presentation (52%). Amoxicillin-clavulanic acid was administered to 36 dogs, a fluoroquinolone to 13 dogs, metronidazole to four dogs, cefalexin to two dogs and gentamicin to one dog. Two dogs had a history of recent vaccination (7 and 17 days prior to diagnosis respectively).

### Diagnosis

Using the ACVIM consensus, 42 dogs had a definitive diagnosis of IMHA (40%), 50 dogs had a diagnosis supportive of IMHA (48%) and the remaining 12 dogs had a diagnosis suspicious for IMHA (12%). Twenty-one dogs belonging to the “suspicious” category were excluded because other causes could potentially explain the anaemia despite documentation of spherocytosis, positive agglutination or positive Coombs’ test.

Spherocytosis was documented in 81 dogs (78%). Information regarding the slide agglutination test were available in 96 cases. Persistent agglutination after 1:4 dilution was documented in 68 cases (71%). Two dogs presented with mildly positive agglutination that did not persist after dilution. The remaining 26 cases had a negative agglutination. Coombs’ test results were available in 51 dogs and were positive in 32 cases (63%). Ten dogs were diagnosed with nrIMA (10%). Of these, five had bone marrow examination performed that documented erythroid hyperplasia.

**Fig. 1:**
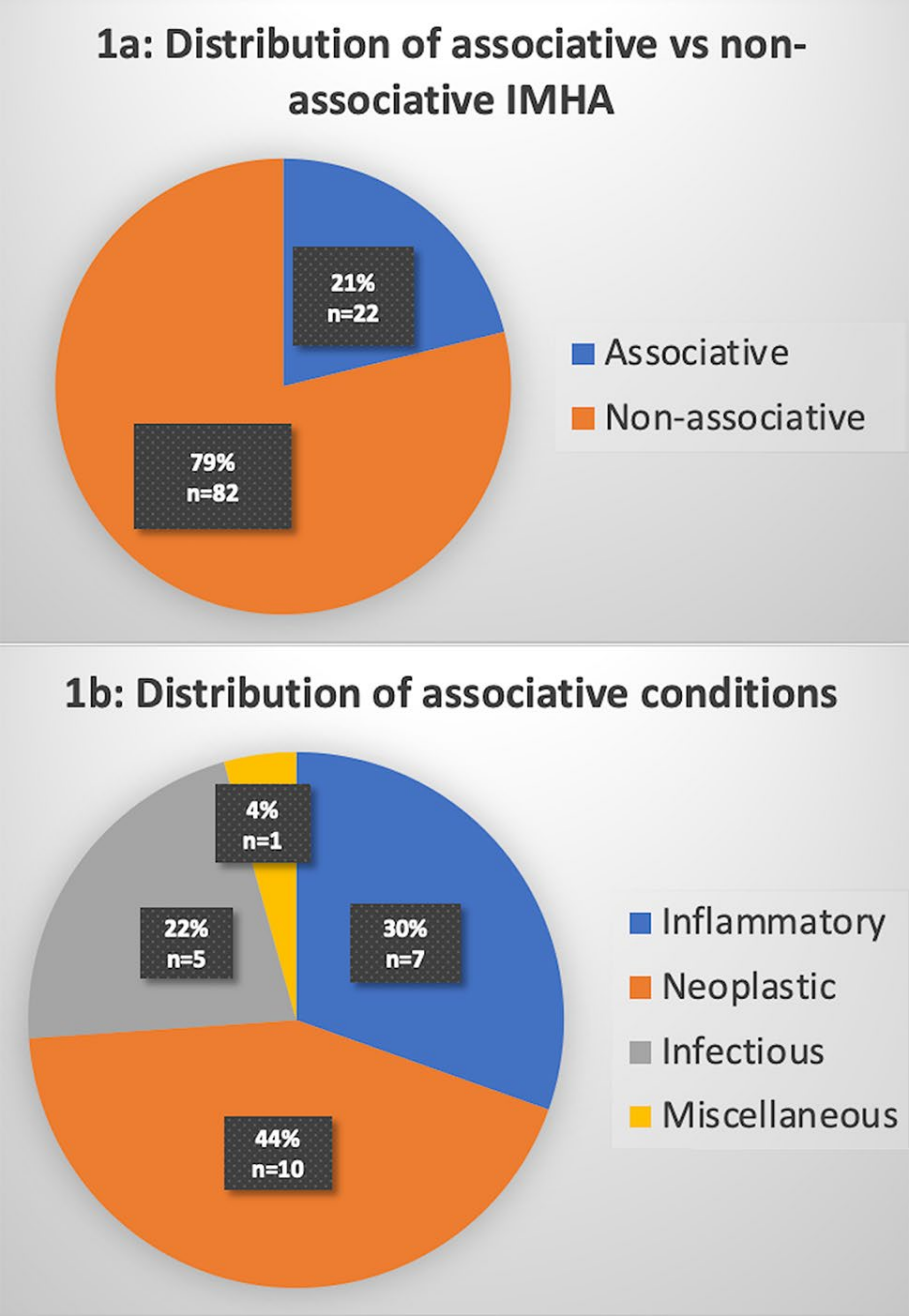
Distribution of IMHA according to the presence and type of associative conditions. **a**: Presence or absence of associative conditions. **b**: Type of associative conditions

Associative conditions were found in 22 dogs (21%). The evidence for IMHA was considered diagnostic in nine cases, supportive in nine cases and suspicious in four cases. The associative conditions were neoplastic in 10 dogs (44%), inflammatory in seven dogs (30%), infectious in five dogs (22%), and miscellaneous in one dog (Fig. 1; Table 1). Three dogs had two concurrent associative conditions (pancreatitis and vasculitis, sino-nasal aspergillosis and aspiration pneumonia respectively).

**Table 1:** Subtypes of conditions in 22 dogs with associative IMHA

Infectious	Inflammatory	Neoplastic	Miscellaneous
Sino-nasal aspergillosis (*n* = 1)	Pancreatitis (*n* = 7)	Multicentric nodal lymphoma (*n* = 2, not further characterized)	Hepatopathy of unknown origin (*n* = 1)
Aspiration pneumonia requiring antimicrobial (*n* = 2)	Suspected vasculitis (*n* = 1)	Acute leukaemia (*n* = 2, not further characterized)	
Cholecystitis (*n* = 1)		Confirmed pulmonary carcinoma (*n* = 1)	
Prostatitis (*n* = 1)		Mammary carcinoma (*n* = 2, not further characterized)	
Bite wound infection (*n* = 1)		Neoplasia that were suspected based on imaging but not cytologically/histologically investigated (pulmonary carcinoma *n* = 1, cranial mediastinal mass *n* = 1, abdominal mass with suspected liver metastasis *n* = 1)	

### Clinicopathological and imaging results

Median haematocrit on presentation was *0.16 L/L (0.05–0.39 –* Table 2). Median reticulocyte count was 111.7* × *10^9/L (2.7–664) and reticulocytosis was documented in 71.3% of the cases (Table 2).

Thrombocytopenia and severe thrombocytopenia were documented in 28% and 12% of the cases, respectively (Table 2).

**Table 2:** Haematological results in a population of 104 dogs with IMHA

Laboratory results	Median	Reference interval (RI)	Range	Above RI Number (%)	Below RI Number (%)
**Haematocrit** (L/L)	0.16	0.37–0.55	0.05–0.39		103 (99)
**Reticulocyte** (10^9^/L)	111.7	0–60	2.7–664	62 (71.3)	
**Leucocytes** (10^9^/L)	23	6–17	4-116	67 (64)	
**Neutrophils** (10^9^/L)	16.7	3-11.5	1.6–98.2	73 (70)	
**Band neutrophils** (10^9^/L)	0.5	0	0-12.3	55 (53)	
**Lymphocytes** (10^9^/L)	1.7	1-3.6	0-10.5	22 [22]	
**Monocytes** (10^9^/L)	1.45	0-1.35	0-10.8	54 (53)	
**Platelets** (10^9^/L)	202	150–500	1-13333		< 150: 29 [28] < 50: 11 [12]

Median bilirubin concentration was 14.2 µmol/L (1.2-710.7) and 62 dogs (61%) presented with hyperbilirubinaemia (Table 3). There were more dogs presenting with increased urea concentration alone: 45 dogs (45%) compared to dogs with increased urea and creatinine concentrations: 3 dogs (3% - Table 3). Serum albumin concentration was available in 78 dogs and hypoalbuminaemia was documented in 8 dogs (10% - Table 3).

The results of the remaining parameters were available for all 104 dogs. The CHAOS score could be retrospectively calculated in 78 dogs: the median score was 3 (0–7).

**Table 3:** Biochemical results in a population of 104 dogs with IMHA

Laboratory results	Number of dogs with data available	Median	RI	Range	Above RI Number (%)	Below RI Number (%)
**Urea** (mmol/L)	104	8.2	3.6–8.6	2.9–23.6	45 (45)	
**Creatinine** (µmol/L)	104	66	20–120	31–209	3 [3]	
**Bilirubin** (µmol/L)	104	14.2	0–10	1.2-710.7	62 (61)	
**Cholesterol** (mmol/L)	104	5.8	3.2–6.5	2.6–16.9	42 (42)	7 [7]
**Albumin** (g/L)	78	29.8	25–38	18-29.2		8 [10]

Prothrombin and activated partial thromboplastin times were assessed in 59 dogs (57%) including all cases with concurrent thrombocytopenia. There was a clinically-relevant prolongation of clotting times in one case. This dog was euthanized shortly after presentation without further investigation.

Vector-borne disease testing was performed in 33 dogs (32%) with 31 dogs undergoing serological testing for *Ehrlichia*, *Anaplasma* and *Borrelia* and two dogs undergoing blood PCR for *Babesia spp*, *Ehrlichia* and *Anaplasma*. Results were negative for all dogs tested. *Angiostrongylus vasorum* testing was performed in 19 dogs (18%) and was negative in all dogs. Urinalysis was performed in 82 dogs (79%). Urine culture was performed in 13 cases. Bacteriuria (*E. coli*) was identified in one dog diagnosed with prostatitis.

Thoracic radiographs were performed in 94 dogs (90%). Only 6 dogs had clinically relevant findings: a lung mass was found in two dogs, aspiration pneumonia diagnosed in one dog, thromboembolism was suspected in one dog with acute respiratory distress, and radiographic features of pulmonary hypertension were suspected in 2 dogs. Ninety-six dogs (92%) had an abdominal ultrasound performed. Clinically-relevant findings were identified in nine dogs (13%) and included signs of pancreatitis (four dogs), prostatitis (one dog), cholecystitis (one dog), organomegaly and lymphadenopathy in one dog diagnosed with multicentric lymphoma and one dog with acute unclassified leukaemia. In the remaining dog, portal and splenic thrombi were identified. Thoraco-abdominal CT scan examination was performed instead of thoracic radiographs and abdominal ultrasound in one dog and was unremarkable.

### Treatment

Blood transfusions were administered in 67 dogs (64%). Fifteen and 62 dogs (15% and 60%) received a blood transfusion prior to referral and while hospitalized, respectively. The median number of blood transfusion was 1 (0–4). Twenty-four dogs received more than one blood transfusion (23%). Regarding the blood products, 34 dogs (51%) received packed red blood cells (pRBC). Twelve dogs received a combination of whole blood and pRBC (18%). Three dogs (4%) received whole blood only. The blood product was unknown for the remaining 18 dogs (27%).

Steroid therapy was administered in 98 dogs (94%). For the six remaining dogs, steroid therapy was not initiated because of death shortly after admission in the hospital (four dogs). Due to the concerns for severe side effects, mycophenolate mofetil was initiated as sole therapy in 2 giant breed dogs. The type and dose of glucocorticoid administered were not standardized. Most dogs (92 dogs out of the 98 that received glucocorticoids) received initially dexamethasone therapy (median 0.3 mg/kg, minimum – maximum [Min – Max] 0.2–0.4) followed by prednisolone therapy (median 1.9 mg/kg, [Min – Max] 1.0-2.2). For the remaining six dogs, all diagnosed with nrIMA, prednisolone was the initial therapy administered.

Nine dogs weighing more than 30 kg received a prednisolone dose based on surface area (40-50mg/m^2^) which resulted in a lower dose per body weight. The five dogs that were diagnosed with bacterial infection received a lower dose of dexamethasone and prednisolone. There was no change in initial steroid therapy type or dose over the study period.

During hospitalization, two or more immunosuppressants were combined in 62 dogs (60%), with 55 dogs receiving two immunosuppressants (56%), and the remaining seven dogs receiving three immunosuppressants (7%). Further information regarding the type of second-line immunosuppressant (SLI) used over time is provided in Table 4. The combination of three immunosuppressants included prednisolone, azathioprine and ciclosporine (three dogs), and prednisolone, mycophenolate mofetil and ciclosporine (four dogs). In addition, two dogs that were receiving prednisolone and ciclosporine also received human intravenous immunoglobulin. The reasons for and timing to initiation of a second-line or third-line immunosuppressant were not documented in the medical record.

**Table 4:** Second-line immunosuppressants used over time

Time period	SLI Number of dogs (%)	Azathioprine Number (%)	Ciclosporine Number (%)	Mycophenolate mofetil Number (%)
2002–2020	55 (56)	30 (55)	16 [29]	9 [16]
2002–2013	21 (38)	21 (100)	0 (0)	0 (0)
2014–2018	15 [27]	5 [33]	4 [27]	6 (40)
2019–2020	19 (35)	4 [21]	12 (63)	3 [16]

Seventy-eight dogs (75%) received anti-thrombotic medication. Among the 26 dogs that did not receive anti-thrombotic treatment, three dogs had thrombocytopenia with overt signs of bleeding and three dogs died on the day of presentation. Fifteen dogs (14%) received aspirin and 63 dogs (61%) received clopidogrel. All the dogs receiving aspirin were diagnosed before 2014. One dog with neurological signs suspected secondary to a thromboembolism was treated with a combination of clopidogrel and low molecular weight heparin.

### Follow-up and outcome

A complete follow-up until death or until the end of the study was available for 70 dogs (67%). Twenty-seven dogs out of 70 were known to be alive at the end of the study (39%). For the remaining 34 dogs, the median time at which the follow-up was lost was 63 days (1-1635).

Ninety dogs survived to discharge (87%). Out of the 14 dogs that died within the hospital, seven were euthanized and seven died spontaneously. A relapse was documented in six out of the 90 ninety dogs that survived to discharge (7%). The median time to relapse was 157 days (30–692). No dogs were documented to relapse more than once.

**Fig. 2:**
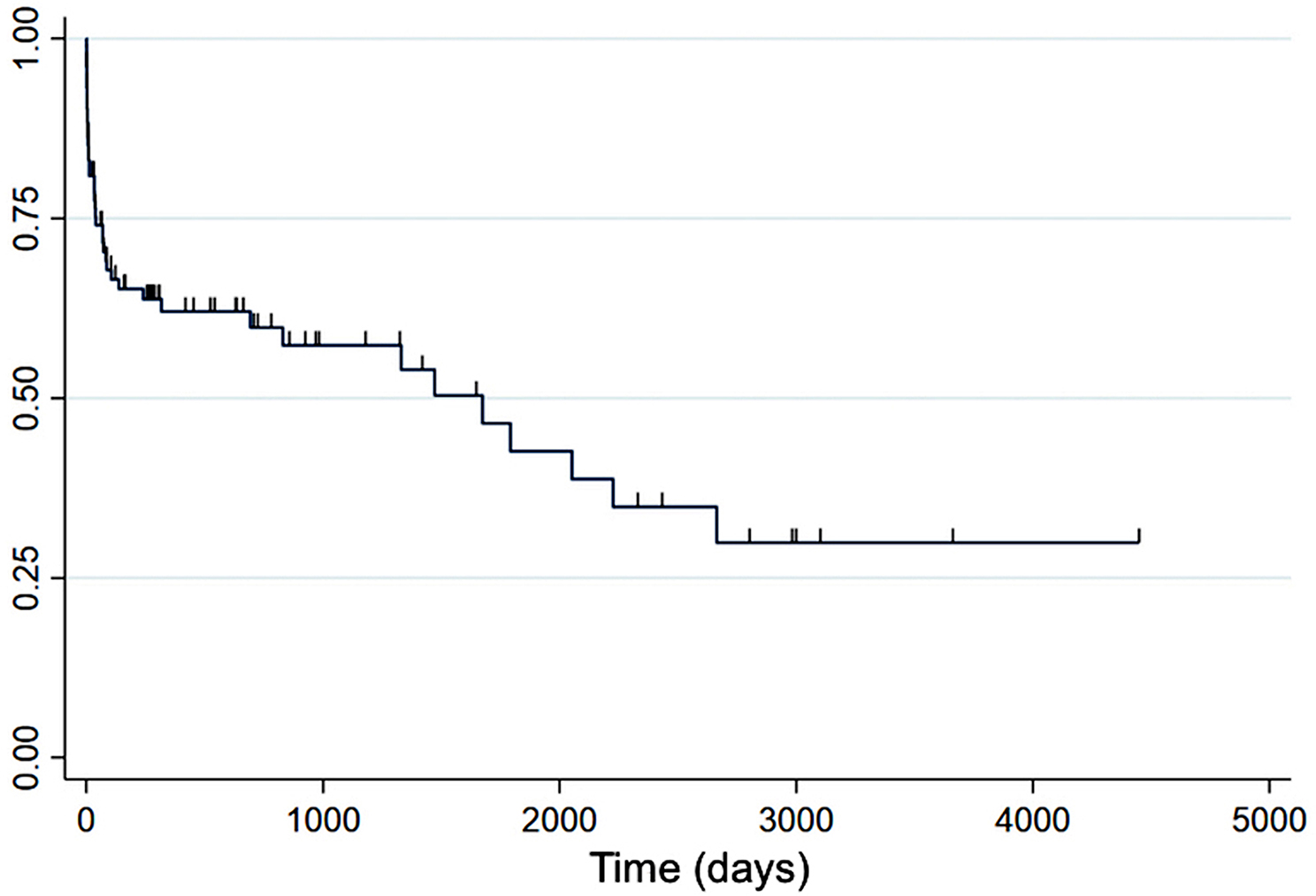
Kaplan Meier survival curve for overall survival. Steep decrease in survival within the first one-three months of diagnosis. Y-axis: survival rate. Censored observations are indicated by tick marks

After excluding dogs with associative conditions, the mortality rate at discharge, and at one, three, six and 12 months was 13%, 16%, 31%, 34% and 38% respectively (Fig. 1). Excluding dogs that died within three months of diagnosis, the median survival time was 2664 days (Fig. 2). The cause of death was not reported in the majority of the cases (24 out of 43 cases, 56%). Four deaths were documented to be non-IMHA related (9%). Deaths were directly attributed to IMHA in 15 out of the 43 deaths (35%) and occurred before discharge (8 dogs), within one month of diagnosis (5 dogs) and within three months (2 dogs). Of the IMHA-related deaths, nine were attributed to a thromboembolic event (60%) and the remaining six deaths were attributed to lack of response to treatment or clinical deterioration (40%). A definitive diagnosis of thromboembolism was reached in five dogs either by post-mortem examination (four dogs) or by imaging (one dog). A tentative diagnosis of thromboembolism was obtained in four dogs (two with acute respiratory distress not explained by other causes and with radiographic or echocardiographic evidence of pulmonary hypertension, and two with acute neurological signs for which advanced imaging was not performed).

The variables assessed for association with survival in the univariable analyses are summarized in Table 5.

**Table 5:** Variables assessed for association with survival in the univariable analysis. Variables with *P* value below 0.2 were included in the multivariate model are indicated with *. Variable with *P* value below 0.05 are indicated with **

Variable	Hazard ratio	95% CI	*P* value
**Age**	1.140	1.031–1.261	0.01*
**Sex (male)**	1.093	0.560–2.132	0.80
**Body weight** **(< 10 kg**,** 10–25 kg**,** > 25)**	1.758 1.848	0.803–3.848 0.767–4.446	0.16* 0.17*
**Increased urea concentration**	1.530	0.833–2.808	0.17*
**Increased creatinine concentration**	2.728	0.649–11.46	0.17*
**Increased bilirubin concentration**	2.204	1.049–4.632	0.04**
**Increased cholesterol concentration**	1.623	0.885–2.977	0.12*
**Decreased albumin concentration**	1.378	0.478–3.968	0.55
**Haematocrit**	0.962	0.919–1.008	0.11*
**Reticulocytes**	0.997	0.995–0.999	0.04**
**Leucocytosis**	0.996	0.979–1.014	0.69
**Neutrophilia**	0.992	0.969–1.014	0.47
**Increased band neutrophils**	0.911	0.498–1.665	0.76
**Lymphopenia**	1.725	0.925–3.218	0.09*
**Monocytosis**	0.744	0.407–1.360	0.34
**Thrombocytopenia**	1.928	1.027–3.618	0.04**
**Severe thrombocytopenia**	1.684	0.708–4.007	0.24
**CHAOS score**	1.453	1.176–1.796	0.001**
**Receiving a blood transfusion**	1.231	0.945–1.604	0.12*
**Number of blood transfusion**	1.289	0.877–1.893	0.19*
**Second-line immunomodulator**	1.688	0.861–3.308	0.13*
**Third-line immunomodulator**	1.793	0.752–4.272	0.19*
**Use of azathioprine**	1.170	0.633–2.162	0.62
**Use of cyclosporine**	1.437	0.674–3.064	0.35
**Use of mycophenolate**	1.470	0.676–3.196	0.33
**Relapse**	1.44	0.515–4.054	0.49

Based on univariable analyses, a higher reticulocyte count was significantly associated with a lower risk of death (HR = 0.997, 95% CI: 0.995–0.999, *P* = 0.04). The bilirubin concentration and the platelet count were not associated with survival (*P* = 0.49 and *P* = 0.92). However, dogs with thrombocytopenia and hyperbilirubinaemia were more likely to die compared to non-thrombocytopenic dogs and dogs with normal bilirubin concentration (HR = 1.93, 95% CI: 1.03–3.63, *P* = 0.04; HR = 2.2, 95% CI: 1.05–4.6, *P* = 0.04 respectively). The higher the CHAOS score the less likely dogs were to survive discharge (HR = 1.45, 95% CI: 1.2–1.80, *P* = 0.001). The remaining variables with *P* value below 0.2 were included in the multivariable analysis: age, increased urea and creatinine concentrations, hypercholesterolaemia, hypocholesterolaemia, haematocrit, lymphopenia, receiving a blood transfusion, the number of blood *transfusions* received, and the use of second-line and third-line immunosuppressants.

**Fig. 3:**
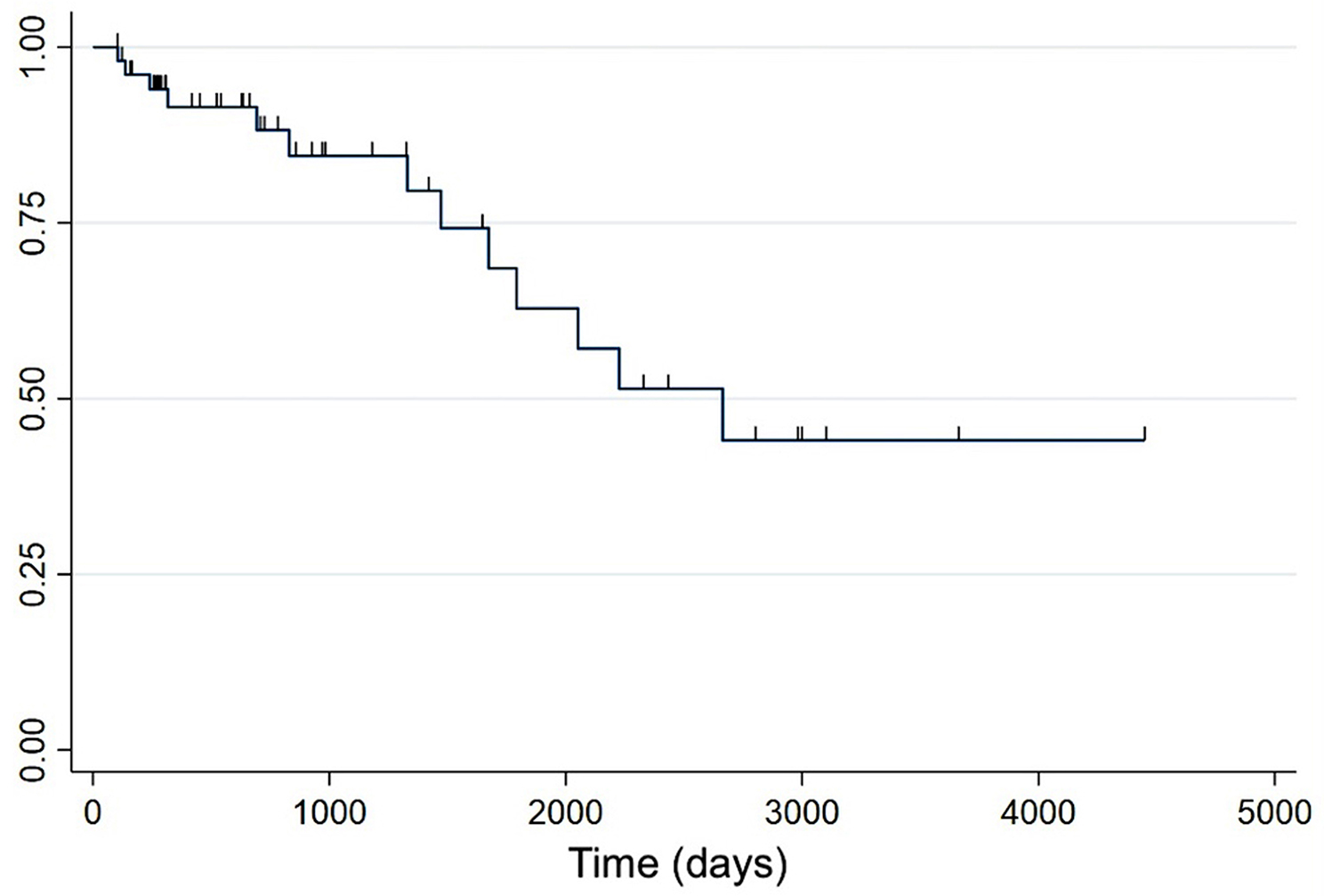
Kaplan-Meier survival curve for long-term survival excluding dogs that died within three months of diagnosis. Y-axis: survival rate. Censored observations are indicated by tick marks

**Fig. 4:**
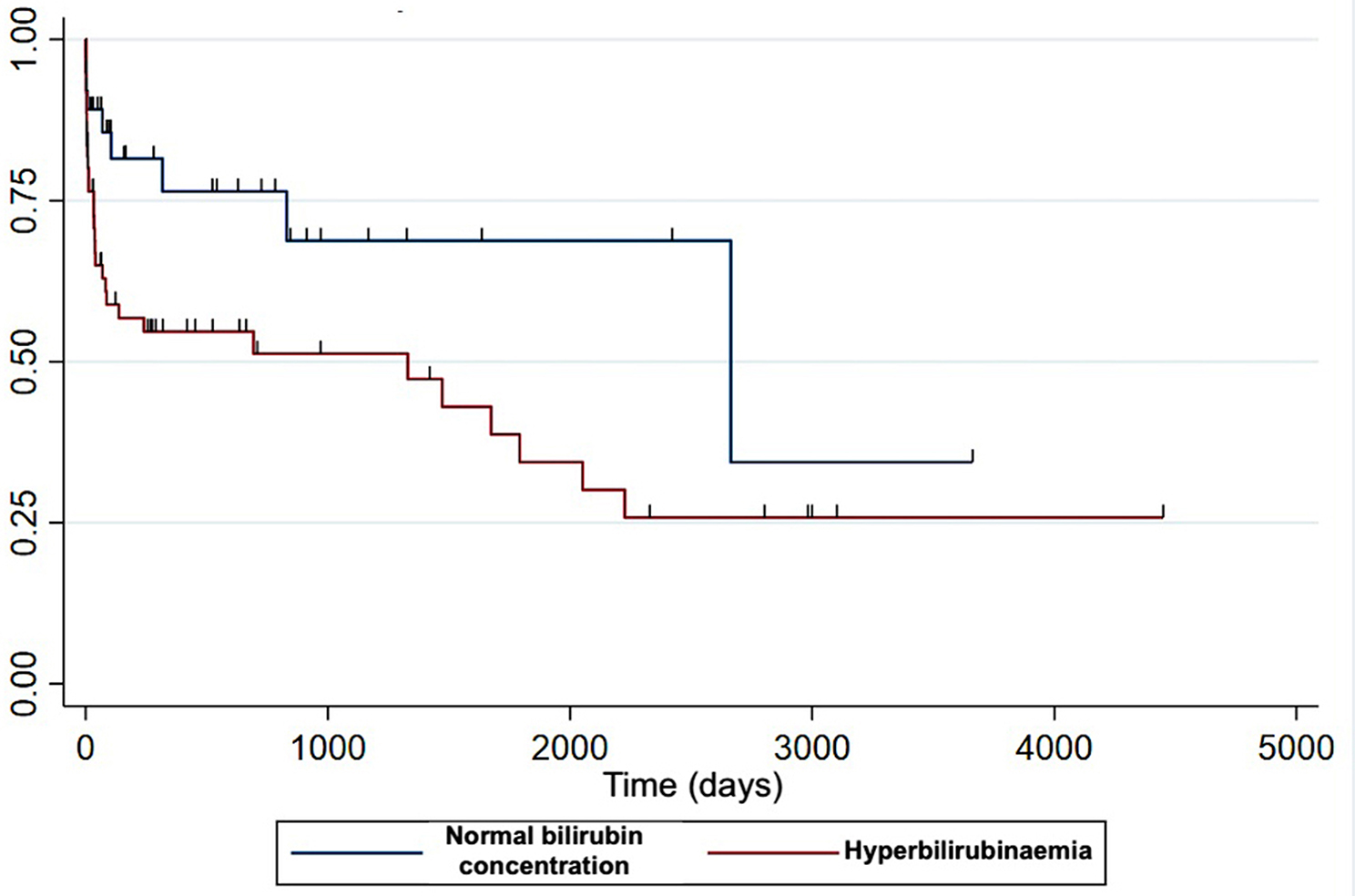
Kaplan-Meier survival curves depending on presence or absence of hyperbilirubinaemia. Decreased likelihood of survival in dogs with hyperbilirubinaemia compared to non-hyperbilirubinaemic dogs. Y-axis: survival rate. Censored observations are indicated by tick marks

Based on the multivariable analysis, only three variables were independently associated with survival: age, thrombocytopenia and hyperbilirubinaemia (Figs. 3 and 4). Controlling for age at diagnosis, thrombocytopenic dogs were 2.2 times more likely to die compared with non-thrombocytopenic dogs (HR = 2.2, 95%CI: 1.1–1.4, *P* = 0.02). Dogs with increased bilirubin concentration were 2.5 times more likely to die compared with dogs with normal bilirubin concentration (HR 2.5, 95% CI [1.1–5.6], *P* = 0.021).

## Discussion

To the best of our knowledge, this study is the first to report clinical presentation, long-term outcome and prognostic factors in a specific population of 104 dogs diagnosed with IMHA in Ireland using the ACVIM consensus statement algorithm. The mortality rate at one and three months was 16 and 31% respectively for dogs with non-associative IMHA. Excluding dogs that died within three months of diagnosis and dogs with associative conditions, the median survival time was 2664 days. The relapse rate was 7%. Despite the extensive study period, the survival rate did not improve over time. In the multivariable analysis, dogs with hyperbilirubinaemia and thrombocytopenia were more likely to die compared to dogs with normal bilirubin concentration and normal platelet count.

In prior studies, thrombocytopenia is regularly identified a negative prognostic factor [11, 12, 14, 22]. Thrombocytopenia was identified in 28% of the cases in the present study. Dogs with IMHA commonly display varying magnitude of thrombocytopenia, which may be consumptive when related to disseminated intra-vascular coagulation (DIC) or thromboembolic events, or may be due to concurrent immune-mediated platelet destruction (Evans syndrome) [1–3, 23]. Irrespective of its pathophysiology, thrombocytopenia either reflects severe complications associated with IMHA or may directly contribute to mortality when overt bleeding occurs, explaining why it is a consistent negative prognostic indicator for canine IMHA.

Hyperbilirubinaemia or icterus has been consistently identified as a negative prognostic factor [6, 7, 9, 12, 13, 15, 16, 22]. It was documented in 61% of the cases in the present cohort. Hyperbilirubinaemia in IMHA is due to a combination of haemolysis and likely decreased hepatic uptake, suspected to be related to hypoxaemia or thromboemboli-induced acute hepatopathy [1, 2, 24]. Similar to thrombocytopenia, the presence of hyperbilirubinaemia likely reflects a more severe or acute course of the disease, which probably explains why it is consistently identified as a negative prognostic indicator.

The signalment of this population was similar to the previously described population of dogs with IMHA. The median age at time of diagnosis in this study was 6.5 years, similar to prior studies [1]. There was also a high number of female and female neutered dogs (71% and 47% respectively). Females have been documented to be over-represented in prior studies, as well as neutered females and neutered dogs [12, 22, 25, 26]. Cocker spaniel, shih tzu and springer spaniel were among the most common breeds in the present study. These three breeds have been documented to be over-represented in IMHA [9, 11, 13, 24, 27].

The median duration of clinical signs prior to presentation was 4 days. This is in agreement with previous studies reporting a median of 3–6 days, and a duration of less than 3 days in 83% of dogs [6, 14, 25]. However, some dogs had a longer duration of clinical signs prior to presentation. This may be due to prior steroid therapy resulting in a suboptimal or intermittent control of the clinical signs, thus delaying time to referral. Indeed, the dog for which the clinical signs lasted for 90 days had a partial response to steroids prior to referral. Alternatively, this may be related to the fact that 10% of the included dogs were diagnosed with non-regenerative anaemia. Most dogs (7/10) were attributed a probable diagnosis of IMHA according to the ACVIM Consensus algorithm. Non-regenerative anaemia has a more insidious onset of clinical signs [28]. Therefore, the inclusion of these cases likely contributed at least partly to the reported prolonged duration of the clinical signs for some dogs.

Unsurprisingly, the most common clinical signs were unspecific (e.g., lethargy, decreased appetite), as previously described [1]. Likewise, gastrointestinal signs were documented in 28% of the cases, similar to prior reports [14, 25, 27]. The gastrointestinal signs are thought to be due to gastrointestinal or extra-gastrointestinal organ hypoxia (e.g., pancreas, kidney, liver) [23]. Fever was documented less frequently in the present study compared to prior data (23% compared to 46%) [14, 29]. This may be due to the high percentage of dogs having received steroid therapy prior to presentation (67%).

The use of the ACVIM consensus statement criteria allowed standardized diagnosis of IMHA. Almost half of the included cases were classified as diagnostic for IMHA. The diagnosis was deemed “supportive” in 48% of the cases, and the diagnosis was “suspicious” in only 12% of cases. The low proportion of suspicious diagnosis reflects the fact that these cases were carefully reviewed, not only using the ACVIM consensus statement criteria but also the available history and follow-up to determine whether the level of evidence was adequate enough to support inclusion. Therefore, IMHA was confidently diagnosed in the present study’s population. However, prior to presentation, 67% of dogs had received steroids, and blood transfusion had been administered in 15% of dogs. Steroid therapy can lead to false-negative results for IMHA investigation (e.g., resulting in decreased spherocytes number, negative agglutination, negative Coombs’ test) [20]. Conversely, blood transfusions can lead to false-positive results for IMHA investigation (spherocytosis, positive agglutination, signs of haemolysis) [20]. It is therefore possible that some cases were under- or over-diagnosed due to the confounding effect of prior therapy.

Associative causes were identified in 21% of the cases, similar to previously published data [14, 23]. Imaging was not performed in nine cases. Among these nine dogs, two had associative causes diagnosed by another method; haematopoietic malignancy by. cytology (one dog) and blood smear examination (one dog). A third dog died quickly after admission, preventing imaging testing but underwent post-mortem examination which did not identify an associative cause. The diagnostic yield of thoracic radiographs and abdominal ultrasound to identify associative causes is reported to be low [30]. Therefore, it seems less likely that associative causes were missed in the remaining six cases. However, it cannot be ruled out.

Regarding immunosuppressive therapy, azathioprine was the most commonly used second-line immunosuppressant overall. However, there was a change in SLI use over time (Table 4). This may be due to the fact that, during the study period, the benefit of azathioprine therapy in IMHA was challenged [6]. Other explanations include increased availability for immunosuppressants with quicker onset of action and less severe side effects [31]. The reason for favouring the use of a specific SLI was not specified in the medical records. Therefore, the preference for ciclosporine may have been related to clinician’s experience, drug availability, or other reasons. Irrespective of this, in the multivariable survival analysis, survival rates were similar regardless of the use of a SLI and regardless of the type of immunosuppressant used.

Prior studies reported similar mortality rates comparable to our cohort: 24% vs. 13% at discharge and 32.3% vs. 16% at one month, 30.4% vs. 31% at three months, 27.3% vs. 34% at six months and 69% vs. 62% at 12 months respectively [6, 7, 10, 14, 23, 32]. Excluding dogs that died within three months and dogs with associative conditions, the median survival time was 2664 days. As previously reported, most dogs that survive the first months following diagnosis have a prolonged survival [10]. Providing owners with information regarding positive outcome in survivor dogs may help them to commit emotionally and financially, which is required for optimal IMHA management.

Survival did not improve over the extensive time of the study period: 18 years, despite changes in management such as type of SLI preferentially used, increased use of thromboprophylaxis over time, and regardless of the number of blood transfusions received. The exclusion of a high number of dogs, and particularly the ones diagnosed from 2002 to 2013, could potentially have biased the data towards over-representation of longer survival, and thus influenced the assessment of its improvement over time.

Thromboembolism is a well-documented life-threating complication of IMHA with a frequency ranging from 16.6 to 80% [1, 3, 23, 33, 34]. 60% of deaths were attributed to thromboembolic events in the present study. This relatively high percentage may be explained by the thromboprophylactic management, as only 63% of the dogs diagnosed with IMHA received anti-thrombotic medication between 2002 and 2013. Practice changed over the years due to higher awareness of thromboembolic complications. Indeed, the use of thromboprophylaxis increased over time during the study period. In the group of dogs diagnosed between 2002 and 2013, 24 out of 38 dogs of dogs received anti-thrombotic medication (63%). Over this period, there were five deaths related to thromboembolic events. Two dogs were not receiving thromboprophylaxis and two dogs were receiving aspirin therapy only. In the 2014–2018 group, 28 out of 38 dogs received anti-thrombotic drugs (74%). Over this period, there were two deaths related to thromboembolism, including one dog not receiving thromboprophylaxis. Finally, 26 out of 28 of dogs in the 2019–2020 group (93%). There were two deaths related to thromboembolism over this period, with both dogs receiving thromboprophylaxis with clopidogrele.

This illustrates that thromboembolic-related deaths are likely explained by inadequate thromboprophylaxis in the earlier years of the study. Conversely, in the more recent years, thromboembolic-related deaths occurred despite adequate thromboprophylaxis. The interpretation of this finding is likely confounded by the fact that more performant imaging modalities were used in the more recent years, probably enabling to detect more thromboembolic events than in the earlier years.

Complete follow-up was available for 70% of the cases. The median time to loss of follow-up in the remaining cases was 63 days (1-1635). The relapse rate was lower in our study (7%) compared to historical data (13–38%) [14, 22, 27] and compared to a well-designed recent study that reported a relapse rate of 22.9% [10]. It is possible that the different relapse rate is related to different treatment protocols (e.g., duration and type of immunosuppression, use of a second-line immunosuppressant). Alternatively, as 30% of dogs were lost to follow up, it cannot be excluded that some relapses were missed, resulting in underestimation of the relapse rate.

This study has several limitations. Its retrospective nature and its extensive period make it possible that some data were missed or inaccurately reported by owners or veterinarians. It is possible that some cases, particularly the ones deemed only suspicious for IMHA were misclassified despite careful review. Five dogs with non-regenerative anaemia did not have bone marrow examination performed, making it possible that some dogs with pure red cell aplasia or precursor-targeted immune-mediated anaemia were erroneously included.

Additionally, the reason for or the time to escalation of immunosuppressive therapy were not recorded. There was no standardized protocol or determined time points to guide the decision to escalate immunosuppressive therapies. Therefore, immunosuppressive therapy protocols may have been chosen depending on clinicians’ preference or disease severity. This may or may not result in a bias towards administration of SLI to more severe cases, thus complicating the assessment of efficacy of immunosuppressive therapy in the present population.

Regarding the survival analysis, the endpoint was death by all cause and not IMHA-related death. This could also may decrease the relevance of the results, as using parameters associated with disease-related risk of death is more likely to be useful in decision-making in the acute setting.

Despite these limitations, given the number of dogs included, the use of standardized criteria to define IMHA, the use of multivariate analysis and the long-term follow-up available in most cases, this study provides valuable input regarding the clinical presentation, treatment and outcome of canine IMHA in Ireland.

## Conclusion

Excluding dogs that died within three months, the long-term outcome of dogs diagnosed with non-associative IMHA in Ireland was excellent and the relapse rate was low. Thrombocytopenia and hyperbilirubinaemia were independent factors associated with decreased survival in this population. The mortality rate was similar compared to prior studies and did not improve over the extensive study period despite improvement in standard of care. Even if managed according to current standards, IMHA remains a life-threatening condition within three months of diagnosis. A better understanding of the pathogenesis of IMHA is required to help develop more targeted immunosuppressive therapy and thromboprophylaxis, in order to improve its outcome.

**Fig. 5:**
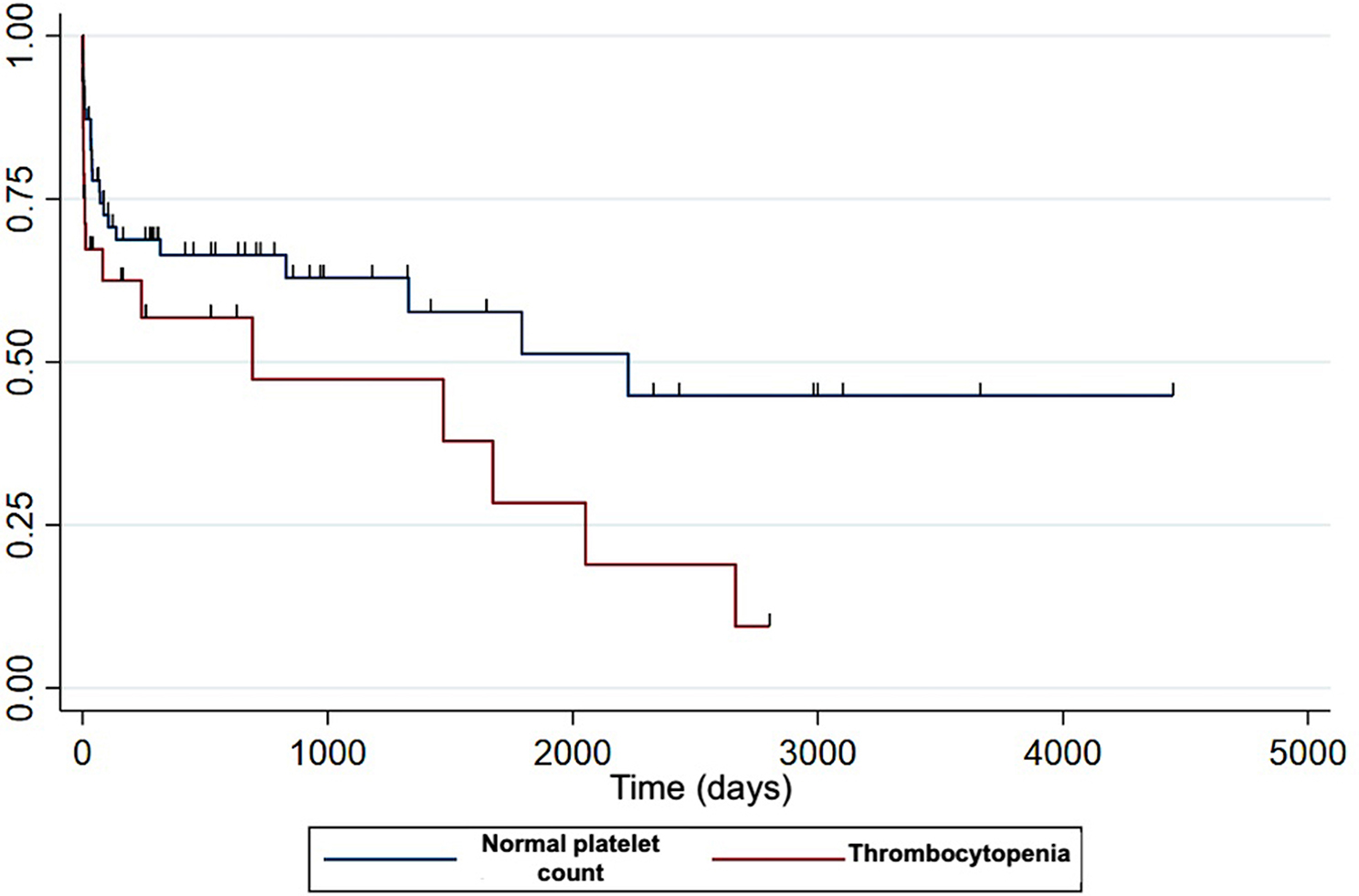
Kaplan-Meier survival curves depending on presence or absence of thrombocytopenia. Decreased likelihood of survival in dogs with thrombocytopenia compared to non-thrombocytopenic dogs. X-axis: time (days)). Y-axis: survival rate. Censored observations are indicated by tick marks

## Data Availability

The dataset used and/or analyzed during the current study are available from the corresponding author on reasonable request.

## Abbreviations

ACVIM: American College of Veterinary Internal Medicine
CHAOS: Canine Haemolytic Anaemic Objective Score
CI: Confidence Interval
DIC: Disseminated Intra-vascular Coagulation
HR: Hazards Ratio
IMHA: Immune-Mediated Haemolytic Anaemia
nrIMA: non-regenerative Mmmune-Mediated Anaemia
pRBC: packed Red Blood Cells
RI: Reference Interval
SAT: Slide Agglutination Test
SLI: Second-Line Immunosuppressant

## References

[1] Piek CJ. Canine idiopathic immune-mediated haemolytic anaemia: a review with recommendations for future research. Vet Q. 2011;31(3):129–41. 22029883 10.1080/01652176.2011.604979

[2] Balch A, Mackin A. Canine immune-mediated hemolytic anemia: pathophysiology, clinical signs, and diagnosis. Compend Contin Educ Vet. 2007;29(4):217–25. 17726851

[3] Goggs R. Therapeutic strategies for treatment of Immune-mediated hemolytic Anemia. Vet Clin North Am Small Anim Pract. 2020;50(6):1327–49. 32814628 10.1016/j.cvsm.2020.07.010

[4] Swann JW, Garden OA, Fellman CL, Glanemann B, Goggs R, LeVine DN, et al. ACVIM consensus statement on the treatment of immune-mediated hemolytic anemia in dogs. J Vet Intern Med. 2019;33(3):1141–72. 30847984 10.1111/jvim.15463 PMC6524099

[5] McAlees TJ. Immune-mediated haemolytic anaemia in 110 dogs in Victoria, Australia. Aust Vet J. 2010;88(1–2):25–8. 20148823 10.1111/j.1751-0813.2009.00537.x

[6] Piek CJ, van Spil WE, Junius G, Dekker A. Lack of evidence of a beneficial effect of azathioprine in dogs treated with prednisolone for idiopathic immune-mediated hemolytic anemia: a retrospective cohort study. BMC Vet Res. 2011;7:15. 21489250 10.1186/1746-6148-7-15 PMC3096914

[7] Goggs R, Dennis SG, Di Bella A, Humm KR, McLauchlan G, Mooney C, et al. Predicting Outcome in dogs with primary Immune-mediated hemolytic Anemia: results of a Multicenter Case Registry. J Vet Intern Med. 2015;29(6):1603–10. 26473338 10.1111/jvim.13642 PMC4864895

[8] Ke SS, Anderson GA, Connolly SL. Evaluation of prognostic indicators for canine primary immune-mediated haemolytic anaemia and application of a scoring system for the determination of prognosis. Aust Vet J. 2015;93(4):93–8. 25817973 10.1111/avj.12302

[9] Reimer ME, Troy GC, Warnick LD. Immune-mediated hemolytic anemia: 70 cases (1988–1996). J Am Anim Hosp Assoc. 1999;35(5):384–91. 10493413 10.5326/15473317-35-5-384

[10] Weingart C, Thielemann D, Kohn B. Primary immune-mediated haemolytic anaemia: a retrospective long-term study in 61 dogs. Aust Vet J. 2019;97(12):483–9. 31454853 10.1111/avj.12875

[11] Ishihara M, Fujino Y, Setoguchi A, Takahashi M, Nakashima K, Ohno K, et al. Evaluation of prognostic factors and establishment of a prognostic scoring system for canine primary immune-mediated hemolytic anemia. J Vet Med Sci. 2010;72(4):465–70. 20009421 10.1292/jvms.09-0283

[12] Carr AP, Panciera DL, Kidd L. Prognostic factors for mortality and thromboembolism in canine immune-mediated hemolytic anemia: a retrospective study of 72 dogs. J Vet Intern Med. 2002;16(5):504–9. 12322697 10.1892/0891-6640(2002)016<0504:pffmat>2.3.co;2

[13] Swann JW, Skelly BJ. Evaluation of immunosuppressive regimens for immune-mediated haemolytic anaemia: a retrospective study of 42 dogs. J Small Anim Pract. 2011;52(7):353–8. 21668886 10.1111/j.1748-5827.2011.01074.x

[14] Piek CJ, Junius G, Dekker A, Schrauwen E, Slappendel RJ, Teske E. Idiopathic immune-mediated hemolytic anemia: treatment outcome and prognostic factors in 149 dogs. J Vet Intern Med. 2008;22(2):366–73. 18346140 10.1111/j.1939-1676.2008.0060.x

[15] Swann JW, Skelly BJ. Systematic review of prognostic factors for mortality in dogs with immune-mediated hemolytic anemia. J Vet Intern Med. 2015;29(1):7–13. 25586014 10.1111/jvim.12514 PMC4858088

[16] Holahan ML, Brown AJ, Drobatz KJ. The association of blood lactate concentration with outcome in dogs with idiopathic immune-mediated hemolytic anemia: 173 cases (2003–2006). J Vet Emerg Crit Care (San Antonio). 2010;20(4):413–20. 20731807 10.1111/j.1476-4431.2010.00551.x

[17] Grundy SA, Barton C. Influence of drug treatment on survival of dogs with immune-mediated hemolytic anemia: 88 cases (1989–1999). J Am Vet Med Assoc. 2001;218(4):543–6. 11229506 10.2460/javma.2001.218.543

[18] Zoia A, Gerou-Ferriani M, Drigo M, Caldin M. Case-control study of plasma mean platelet component concentration and survival analysis for dogs with immune-mediated hemolytic anemia. J Am Vet Med Assoc. 2018;252(11):1384–92. 29772969 10.2460/javma.252.11.1384

[19] Cuq B, Blois SL, Bédard C, Wood RD, Abrams-Ogg AC, Beauchamp G, et al. Serum interleukin 17 concentrations in dogs with immune-mediated hemolytic anemia. J Vet Intern Med. 2021;35(1):217–25. 33219716 10.1111/jvim.15977 PMC7848375

[20] Garden OA, Kidd L, Mexas AM, Chang YM, Jeffery U, Blois SL, et al. ACVIM consensus statement on the diagnosis of immune-mediated hemolytic anemia in dogs and cats. J Vet Intern Med. 2019;33(2):313–34. 30806491 10.1111/jvim.15441 PMC6430921

[21] Robbins R, Viviano KR. Hypocholesterolemia and nonregenerative, suspected immune-mediated, anemia: report of 3 canine cases. Can Vet J. 2017;58(10):1100–4. 28966361 PMC5603918

[22] Weinkle TK, Center SA, Randolph JF, Warner KL, Barr SC, Erb HN. Evaluation of prognostic factors, survival rates, and treatment protocols for immune-mediated hemolytic anemia in dogs: 151 cases (1993–2002). J Am Vet Med Assoc. 2005;226(11):1869–80. 15934255 10.2460/javma.2005.226.1869

[23] McCullough S. Immune-mediated hemolytic anemia: understanding the nemesis. Vet Clin North Am Small Anim Pract. 2003;33(6):1295–315. 14664200 10.1016/j.cvsm.2003.08.003

[24] McManus PM, Craig LE. Correlation between leukocytosis and necropsy findings in dogs with immune-mediated hemolytic anemia: 34 cases (1994–1999). J Am Vet Med Assoc. 2001;218(8):1308–13. 11330619 10.2460/javma.2001.218.1308

[25] Mason N, Duval D, Shofer FS, Giger U. Cyclophosphamide exerts no beneficial effect over prednisone alone in the initial treatment of acute immune-mediated hemolytic anemia in dogs: a randomized controlled clinical trial. J Vet Intern Med. 2003;17(2):206–12. 12683622 10.1111/j.1939-1676.2003.tb02435.x

[26] Miller SA, Hohenhaus AE, Hale AS. Case-control study of blood type, breed, sex, and bacteremia in dogs with immune-mediated hemolytic anemia. J Am Vet Med Assoc. 2004;224(2):232–5. 14736067 10.2460/javma.2004.224.232

[27] Burgess K, Moore A, Rand W, Cotter SM. Treatment of immune-mediated hemolytic anemia in dogs with cyclophosphamide. J Vet Intern Med. 2000;14(4):456–62. 10935898 10.1892/0891-6640(2000)014<0456:toihai>2.3.co;2

[28] Woolhead VL, Szladovits B, Chan A, Swann JW, Glanemann B. Breed predispositions, clinical findings, and prognostic factors for death in dogs with nonregenerative immune-mediated anemia. J Vet Intern Med. 2021;35(1):252–60. 33617109 10.1111/jvim.15986 PMC7848385

[29] Mellett AM, Nakamura RK, Bianco D. A prospective study of clopidogrel therapy in dogs with primary immune-mediated hemolytic anemia. J Vet Intern Med. 2011;25(1):71–5. 21155892 10.1111/j.1939-1676.2010.0656.x

[30] Woodward GM, White JD. The utility of screening diagnostic tests in identifying associative immune-mediated haemolytic anaemia in dogs. Aust Vet J. 2020;98(12):586–90. 32935334 10.1111/avj.13016

[31] Viviano KR, Glucocorticoids. Cyclosporine, azathioprine, Chlorambucil, and Mycophenolate in Dogs and cats: clinical uses, Pharmacology, and Side effects. Vet Clin North Am Small Anim Pract. 2022;52(3):797–817. 35379498 10.1016/j.cvsm.2022.01.009

[32] Swann JW, Skelly BJ. Canine autoimmune hemolytic anemia: management challenges. Vet Med (Auckl). 2016;7:101–12. 30050843 10.2147/VMRR.S81869 PMC6055891

[33] Balch A, Mackin A. Canine immune-mediated hemolytic anemia: treatment and prognosis. Compend Contin Educ Vet. 2007;29(4):230–8. quiz 9. 17726852

[34] Weng J, Levy NA, Abbott HY, Mix JA, Wills RW, Mackin AJ et al. Retrospective analysis of immunosuppressive and anti-thrombotic protocols in nonassociative immune mediated hemolytic anemia in dogs. J Vet Intern Med. 2023. 10.1111/jvim.16652 PMC10061171 36809664

